# Differential symbiotic compatibilities between rhizobium strains and cultivated and wild soybeans revealed by anatomical and transcriptome analyses

**DOI:** 10.3389/fpls.2024.1435632

**Published:** 2024-09-03

**Authors:** Sobhan Bahrami Zadegan, Wonseok Kim, Hafiz Muhammad Khalid Abbas, Sunhyung Kim, Hari B. Krishnan, Tarek Hewezi

**Affiliations:** ^1^ Department of Plant Sciences, University of Tennessee, Knoxville, TN, United States; ^2^ Graduate School of Genome Science and Technology, University of Tennessee, Knoxville, TN, United States; ^3^ Plant Science Division, University of Missouri, Columbia, MO, United States; ^4^ Plant Genetics Research, The United States Department of Agriculture (USDA) Agricultural Research Service, Columbia, MO, United States

**Keywords:** *Bradyrhizobium diazoefficiens*, *Glycine max*, *Glycine soja*, nodulation, RNA-Seq

## Abstract

Various species of *rhizobium* establish compatible symbiotic relationships with soybean (*Glycine max*) leading to the formation of nitrogen-fixing nodules in roots. The formation of functional nodules is mediated through complex developmental and transcriptional reprogramming that involves the activity of thousands of plant genes. However, host transcriptome that differentiate between functional or non-functional nodules remain largely unexplored. In this study, we investigated differential compatibilities between rhizobium strains (*Bradyrhizobium diazoefficiens* USDA110 *Bradyrhizobium* sp. strain LVM105) and cultivated and wild soybeans. The nodulation assays revealed that both USDA110 and LVM105 strains effectively nodulate *G. soja* but only USDA110 can form symbiotic relationships with Williams 82. LVM105 formed pseudonodules on Williams 82 that consist of a central nodule-like mass that are devoid of any rhizobia. RNA-seq data revealed that USDA110 and LVM105 induce distinct transcriptome programing in functional mature nodules formed on *G. soja* roots, where genes involved in nucleosome assembly, DNA replication, regulation of cell cycle, and defense responses play key roles. Transcriptome comparison also suggested that activation of genes associated with cell wall biogenesis and organization and defense responses together with downregulation of genes involved in the biosynthesis of isoprenoids and antioxidant stress are associated with the formation of non-functional nodules on Williams 82 roots. Moreover, our analysis implies that increased activity of genes involved in oxygen binding, amino acid transport, and nitrate transport differentiates between fully-developed nodules in cultivated versus wild soybeans.

## Introduction

Legumes such as soybean (*Glycine max*) and nitrogen fixing bacteria can develop a symbiotic relationship to form specialized organs, the nodules, on plant roots within which bacteria reside and convert the atmospheric nitrogen into ammonia readily available for plants to be used (Wang et al., 2018). Nodule formation is a highly complex process, at the cellular and developmental levels, requiring sophisticated crosstalk between symbiotic partners. At the start, rhizobia sense the flavonoids secreted by the host plant to synthesize the lipochitooligosaccharides (Nod factors) and then, a series of signaling and developmental processes begins, resulting in the formation of successful nodules (Jones et al., 2007). Nod factors are recognized by the Nod factor receptors (NFRs) present at the epidermal cells of root hairs. This perception initiates the formation of curls in root hair cells to reside the rhizobia inside and then, to allow them to form the infection thread growing towards the root cortex (Turgeon and Bauer, 1985; Ardourel et al., 1994). As the infection begins, differentiation occurs in the root cortex cells to form nodule primordia, where rhizobia are released into symbiosomes and being differentiated into nitrogen fixing bacteroids. Under certain conditions, the bacteroids accumulate intracellular carbon polymers like polyhydroxybutyrate (PHB), which seems critical for nitrogen fixation (Trainer and Charles, 2006). These bacteroids are then enclosed in dividing cortex cells, developing into final functional nodules that appear red or pink from inside (Oldroyd and Downie, 2008). These nodules significantly contribute to the soybean growth by providing the required amount of nitrogen.

Various species of *rhizobium*, including slow-growing (e.g., *B. japonicum, B. elkanii, B. liaoningense, B. yuanmingense)* and fast-growing (e.g., *S. fredii)* can associate with soybean and successfully induce functional nodules (Biate et al., 2014; Thilakarathna and Raizada, 2017). These rhizobium species as well as strains/isolates belonging to these species vary in their compatibility with different soybean cultivars, and hence nodulation efficiency and nitrogen fixation activity (Biate et al., 2014). The host range of rhizobia is determined by the molecular recognition between symbiotic partners that involves the activity of (*nod*) and nitrogen fixation (*nif*) genes, which are involved in the production of Nod factors and nitrogen fixation, respectively (Perret et al., 2000; Wang et al., 2018; Walker et al., 2020). In addition, it has been recently reported that the exopolysaccharides (EPS) of *B. diazoefficiens* USDA110 contributes to host specificity with different soybean cultivars (Quelas et al., 2010).

Studies have suggested that the domestication of legumes has affected symbiosis-related traits in legumes, as well as the compatibility between host plants and bacteria. It has been shown that wild legume species can associate with more diverse indigenous soil strains than cultivated legumes (Mutch and Young, 2004; Kim et al., 2014; Chang et al., 2019). As an example, the wild species of soybean (*Glycine soja*) exhibits higher nodulation compatibility in comparison with cultivated soybeans (*Glycine max*) (Chang et al., 2019). This shift is believed to be due to domestication as well as breeding and agricultural practices that might have reduced the composition of rhizobial community and compatibility of legume-rhizobial symbiosis (Werner et al., 2015; Turcotte et al., 2017; Liu et al., 2020). Although these practices have led to a decrease in nodulation–rhizobium compatibility in cultivated soybeans, an enhancement in nodulation efficiency and nitrogen fixation activity has been attained (Muñoz et al., 2016; Liu et al., 2020; Wang et al., 2023). In addition, a recent study has shown that genetic variations located in the regulatory regions of the *GmCRP* (*NopC Related Protein*) gene selected during domestication contributes to increased nodulation efficiency in improved soybean cultivars (Wang et al., 2023).

Analysis of naturally occurring genetic variations and artificially induced mutations resulted in the identification of several genetic loci known as *Rj* or *rj* that control symbiotic specificity in soybean. This includes, for example, the recessive alleles *rj1*, *rj5*, and *rj6*, which restrict nodulation; the recessive locus *rj7*, which causes hypernodulation phenotype; and the dominant alleles *Rj3* and *Rfg1*, which limit nodulation with specific strains (Hayashi et al., 2012; Roy et al., 2020). The soybean immune system and rhizobial type-III effectors such as *Nop* genes also play key role in determining the symbiotic outcome (Roy et al., 2020; Jiménez-Guerrero et al., 2022; Teulet et al., 2022). For example, NopL has been shown to interfere with MAPK signaling and inhibit premature nodule senescence (Zhang et al., 2011). NopM in *Ensifer fredii* NGR234 was found to function as E3 ubiquitin ligase during infection in order to reduce the reactive oxygen species in host plants and promote nodulation (Xin et al., 2012). Recently, it has been shown that NopAA effector has a glycosyl hydrolase activity and functions in cell wall remodeling to promote symbiotic nodulation (Wang et al., 2022; Dorival et al., 2020). However, rhizobial effectors can also be recognized as avirulence proteins negatively impacting nodule formation (Staehelin and Krishnan, 2015). For instance, it has been demonstrated that NopD possess SUMO (small ubiquitin-like modifier) protease activity, induces immunity, and negatively affects nodulation (Xiang et al., 2020).

Our understanding of various stages of nodule development was greatly enhanced by recent transcriptome studies using RNA-sequencing approaches (Brechenmacher et al., 2008; Libault et al., 2009, 2010; Severin et al., 2010; Yuan et al., 2016, 2017; Niyikiza et al., 2020). These studies provided interesting insights into the importance of various biological processes during the course of nodule initiation, formation, development, and senescence. This included, for example, hormone signaling pathways, cell wall biogenesis and modifications, regulation of transcription factor activities, defense and immunity responses, primary and secondary metabolic pathways, transporter functions, and small secretory peptides. The complexity of the cellular programing occurring during soybean nodulation is reflected by the identification of more than 9000 genes that establish nodule transcriptome identity (Niyikiza et al., 2020). In addition, gene network analyses of RNA-seq data pointed to the presence of nodule–specific modules of highly co-regulated soybean genes with potential novel functional roles in nodulation (Zhu et al., 2013; Piya et al., 2023).

Despite the reported experimental data showing differential compatibility between rhizobium strains and both cultivated and wild soybeans, anatomical features and host transcriptome that differentiate between functional or non-functional nodules remain poorly understood particularly at later stages of symbiosis. Studying the transcriptome of functional and non-functional nodules at a late stage of development can provide valuable insights into the molecular mechanisms that contribute to nodule functionality and effective nitrogen fixation. In this study, we examined the anatomical features of functional nodules induced by *Bradyrhizobium diazoefficiens* USDA110 on cultivated and wild soybean plants using light and transmission electron microscopy. We also determined the anatomy of functional and non-functional nodules induced by *Bradyrhizobium* sp. strain LVM105 on *G. soja* and Williams 82 roots, respectively. Furthermore, we have investigated transcriptome reprogramming in 25-day-old nodules induced by USDA110 and LVM105 in Williams 82 and *G. soja* using RNA-seq and determined the biological processes and molecular functions that contribute to nodule functionality.

## Materials and methods

### Plant materials

Seeds of progenitor of soybean (*Glycine soja* Sieb. & Zucc. PI 378683) and soybean (*G. max* L. Merr.) cultivar Williams 82 were used in this study.

### Nodulation assay

Bradyrhizobial strains USDA110 and LVM105 were grown in yeast extract mannitol medium at 30°C on a rotary shaker (150 rpm) for 4-5 days. Soybean seeds (*Glycine max* cv Williams 82 and *Glycine soja* PI 378683) were surface-sterilized in 50% bleach (2.5% NaClO) for 5 minutes. Following this step, the seeds were extensively washed in sterile distilled water to get rid of any residual bleach. About 12-15 seeds were placed on 1% water agar plates and placed in a 30°C incubator for 3 days. Roots of three-day-old seedlings were dipped for 2 minutes in USDA110 or LVM105 cultures that had been diluted to 5 x 10^6^ cells per ml. The soybean seedlings were then transferred to sterile Magenta jars containing vermiculite. Soybean plants were transferred to a growth chamber maintained at a 28°C with a light intensity of 500 µmol of photons m^-2^ sec^-1^, and a 12-h light period. The plants were watered with Jensen’s nitrogen-free solution as needed. Nodules were harvested at 25 days post inoculation (DPI) and either processed for ultrastructural analysis or instantly frozen in liquid nitrogen and stored at -80°C.

### Tissue fixation for light and electron microscopy

For light microscopy observation soybean nodules (25 DPI) were fixed in FAA (formaldehyde:ethanol:acetic acid) and embedded in paraffin according to the previously published procedure (Krishnan et al., 2003). Paraffin-embedded nodules were sectioned using a microtome at 10 μm thickness and stained with hematoxylin and eosin. For transmission electron microscopy soybean nodules were cut into small pieces (2 to 4 mm) using a razor blade and were immediately fixed in 2.5% glutaraldehyde in 50 mM cacodylic buffer, pH 7.2 for 4 hours at room temperature. The tissue samples were post-fixed with 2% aqueous osmium tetroxide for 1 hour at room temperature. Following this step, the samples were rinsed in 50 mM cacodylic buffer (pH 7.2) four times at intervals of 15 minutes. The samples were dehydrated in a graded ethanol series and infiltrated with Spurr’s resin (Electron Microscopy Sciences, PA). Thin sections of nodules were produced using a diamond knife and collected on uncoated nickel grids. The tissues were then stained using 0.5% aqueous uranyl acetate and 0.4% aqueous lead citrate, and finally examined with a JEOL JEM 100B (Tokyo, Japan) electron microscope at 100 kV.

### Phylogenetic analysis

The genome sequences of LMV105 (accession number QZMV00000000) and closely related species were retrieved from the NCBI database. Whole genome-based taxonomic analysis was conducted using the Type Genome Server (TYGS) (Meier-Kolthoff and Göker, 2019). The branch lengths were scaled in terms of the genome blast distance phylogeny approach (GBDP) distance formula d5. The tree was rooted at the midpoint.

### RNA-seq analysis

Total RNA from 25 DPI soybean nodules were isolated using TRIzol^®^ Reagent (Thermo Fisher Scientific, Waltham MA, USA). Genomic DNA was removed by treating total RNA with DNase I. RNA integrity was determined using Agilent 2100 (Agilent, Technologies, CA). mRNA was isolated and used for the construction of non-stranded, nondirectional mRNA libraries. Library concentration was first quantified using a Qubit 2.0 fluorometer (Life Technologies), and then using Agilent 2100. mRNA-seq libraries were multiplexed and sequenced using the Illumina NovaSeq 6000 instrument with 150-bp paired-end reads. Data analysis and identification of differentially expressed genes (DEGs) was determined as previously described by Hawk et al. (2023). Briefly, the quality of raw reads was determined using FastQC (http://www.bioinformatics.babraham.ac.uk/projects/fastqc/). Reads were then mapped against the soybean reference genome (Williams 82 genome assembly v4) using STAR (Dobin et al., 2013). The HTSeq tool was utilized to obtain read counts for all transcripts (Anders et al., 2015). Differential gene expression analysis was performed using DESeq2 package with a false discovery rate (FDR) < 1% and fold-change cutoff of 2 (Love et al., 2014). DEGs were analyzed for Gene Ontology (GO) enrichment using the SoyBase database (https://www.soybase.org/) with a significance cut-off value of 0.01. KEGG pathways enrichment analysis was conducted using KOBAS web server (http://bioinfo.org/kobas) with a corrected P value less than 0.01 was considered as statistically significant (Xie et al., 2011). The R packages, “pheatmap” and “ggplot2” (Ito and Murphy, 2013; Kolde, 2015) were used for data visualization.

## Results

### Phylogenomic analysis

We conducted a whole genome-based taxonomic analysis to assess the taxonomic relationships between *Bradyrhizobium* sp. strain LVM105, *Bradyrhizobium diazoefficiens* strain USDA110 and other *Bradyrhizobium* strains. The phylogenetic tree revealed a close phylogenetic relationship between LVM105 and both *Bradyrhizobium frederickii* (CNPSo 3426) and *Bradyrhizobium nanningense* (CCBAU 53390) (**Supplementary Figure 1**). In contrast, the *Bradyrhizobium diazoefficiens* strain USDA110 exhibited a close phylogenetic relationship with *Bradyrhizobium niftali* (CNPSo 3448) and *Rhizobium lupini* (DSM 30140) (**Supplementary Figure 1**). The analysis also indicated that LVM105 and USDA110 are distantly related (**Supplementary Figure 1**).

### Nodulation phenotype of *Bradyrhizobium diazoe*fficiens USDA110 and *Bradyrhizobium* sp. *strain* LVM105 on *Glycine max* and *Glycine soja*

**Figure 1:**
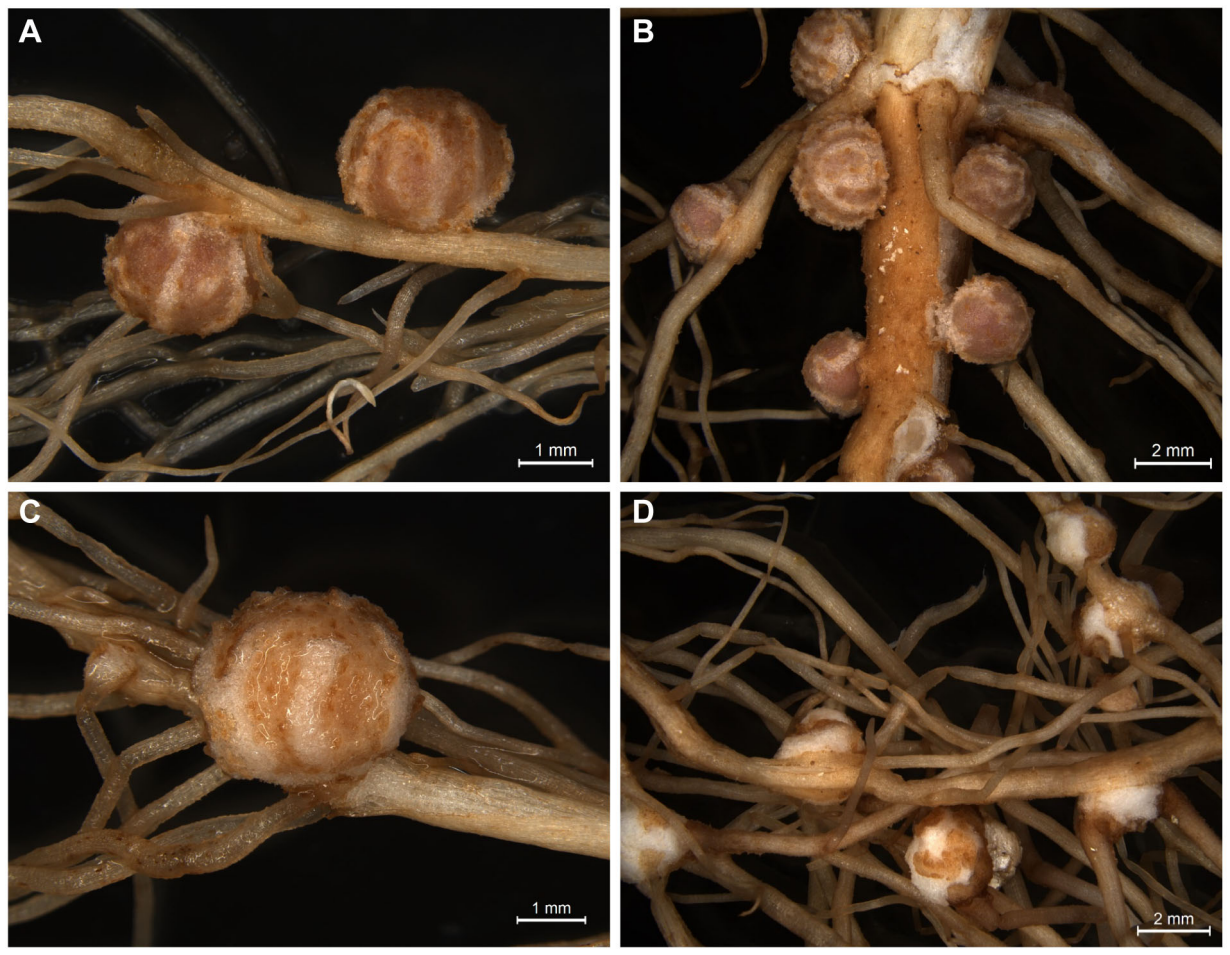
Images of nodules induced by *Bradyrhizobium diazoefficiens* USDA110 and *Bradyrhizobium* sp. strain LVM105 on soybean. **(A, B)**. *Bradyrhizobium diazoefficiens* USDA110 forms nitrogen-fixing nodules on both *Glycine soja* **(A)** and *Glycine max* cv. Williams 82 **(B)**. In contrast, *Bradyrhizobium* sp. strain LVM105 forms nitrogen-fixing nodules only on *Glycine soja* **(C)** and “popcorn” like nodules on *G. max* cv. Williams 82 **(D)**.

*Bradyrhizobium diazoefficiens* USDA110, is the most widely used strain in commercial inoculants for soybean crops. This model strain forms nitrogen-fixing nodules on most commercial soybeans as well as on *Glycine soja*, a wild progenitor of the commercial soybean. In contrast, little is known about the host range of *Bradyrhizobium* sp. strain LVM105. Originally, this strain was isolated from partridge pea (*Chamaecrista fasciculata*), an annual legume of the subfamily *Caesalpinioideae*. The nodulation assays revealed that USDA110 forms nitrogen-fixing nodules on both *G. max* cv Williams 82 and *G. soja* (**Figures 1A, B**). LVM105 efficiently nodulated *Glycine soja* (**Figure 1C**). However, LVM105 lost its ability to form nodules on agronomically ameliorated soybean cultivar such as Williams 82 (**Figure 1D**). LVM105 elicited popcorn nodules (abnormal root proliferations) on the roots of Williams 82 (**Figure 1D**). Our finding that LVM105 is genetically distant from USDA110 and elicits the formation of pseudonodules on Williams 82, prompted us to use this strain over other strains to investigate the anatomical and transcriptome differences between functional and non-functional nodules as detailed below.

### Anatomy of soybean nodules induced by USDA110 and LVM105

**Figure 2:**
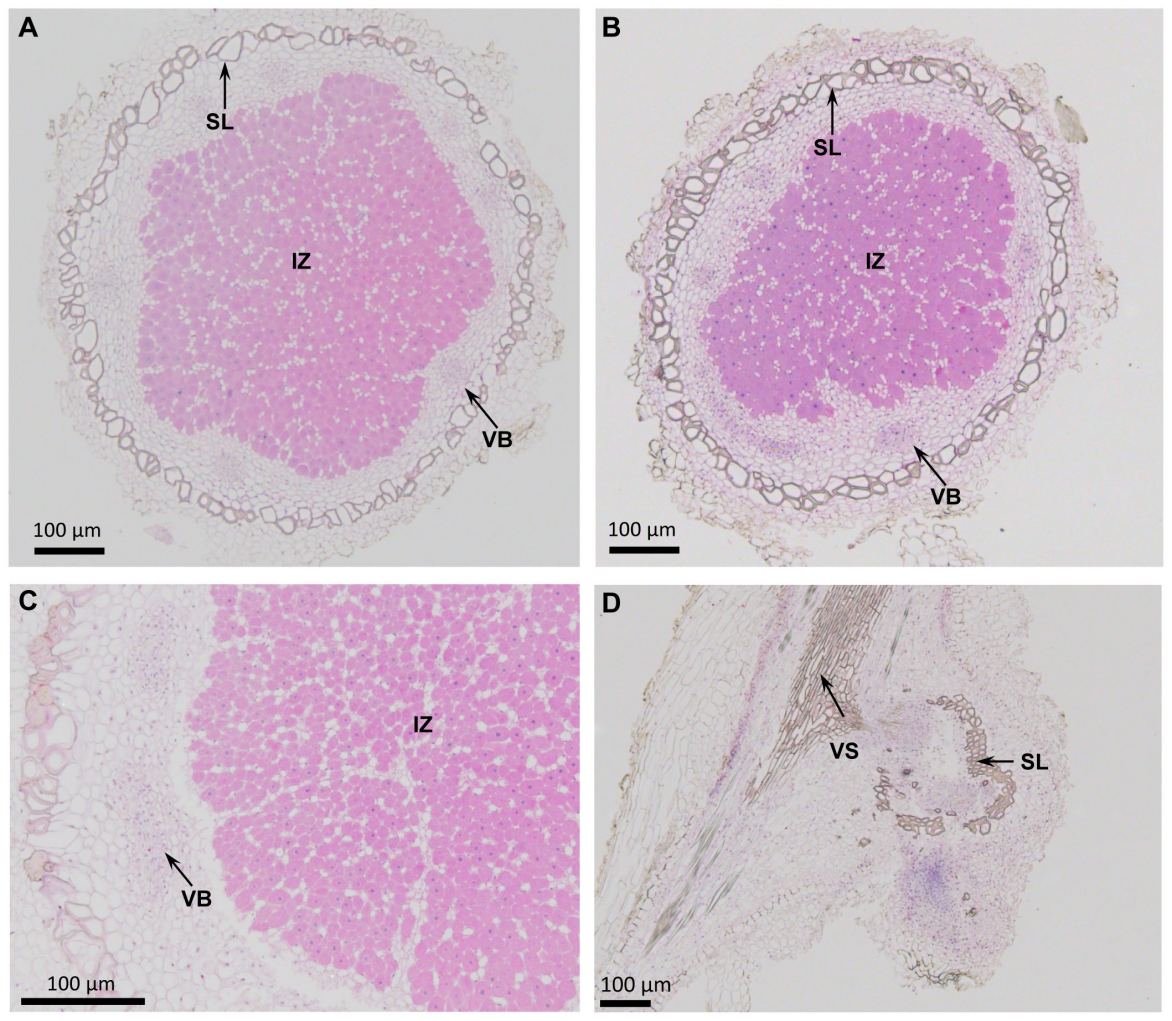
Anatomy of nodules induced by *Bradyrhizobium diazoefficiens* USDA110 and *Bradyrhizobium* sp. strain LVM105 on soybean. **(A, B)** Cross-sections of *Glycine soja* **(A)** and *Glycine max* cv. Williams 82 **(B)** nodules induced by *Bradyrhizobium diazoefficiens* USDA110. **(C, D)** Cross-sections of *G. soja* **(C)** and *G. max* cv. Williams 82 **(D)** nodules induced by *Bradyrhizobium* sp. strain LVM105. Note that infected central region is filled with rhizobia and is surrounded by a scleroid layer **(A-C)**. However, the nodules induced by LVM105 on *G. max* cv. Williams 82 is devoid of rhizobia and lacks the infected central region **(D)**. IZ, infected zone; SL, scleroid layer; VB, vascular bundle; VS, Vascular strand.

A cross-section of 25 DPI soybean nodules elicited by USDA 110, when examined under light microscopy, revealed typical anatomical features of a determinate nodule (**Figures 2A, B**). Both *G. max* cv Williams 82 and *G. soja* nodules contained a large central infected zone. The infected central zone contained cells that were filled with bradyrhizobia along with a few uninfected cells. Surrounding this central zone there is a layer of cells that contain scattered vascular bundles. This layer of cells was surrounded by a prominent sclerenchymatous layer of cells (**Figures 2A, B**). An examination of LVM105–induced nodules in *G. soja* revealed similar anatomical features observed with USDA110–elicited nodules (**Figure 2C**). In contrast, a cross-section of the “popcorn nodules” elicited by LVM105 on the roots of Williams 82 did not reveal any central infected zone, indicating the absence of bradyrhizobia in these structures (**Figure 2D**). A sclerenchymatous layer of thick-walled cells were seen in the central portion of the “popcorn nodules”. Additionally, this layer was surrounded by an outer layer of parenchymatous cells (**Figure 2D**).

**Figure 3:**
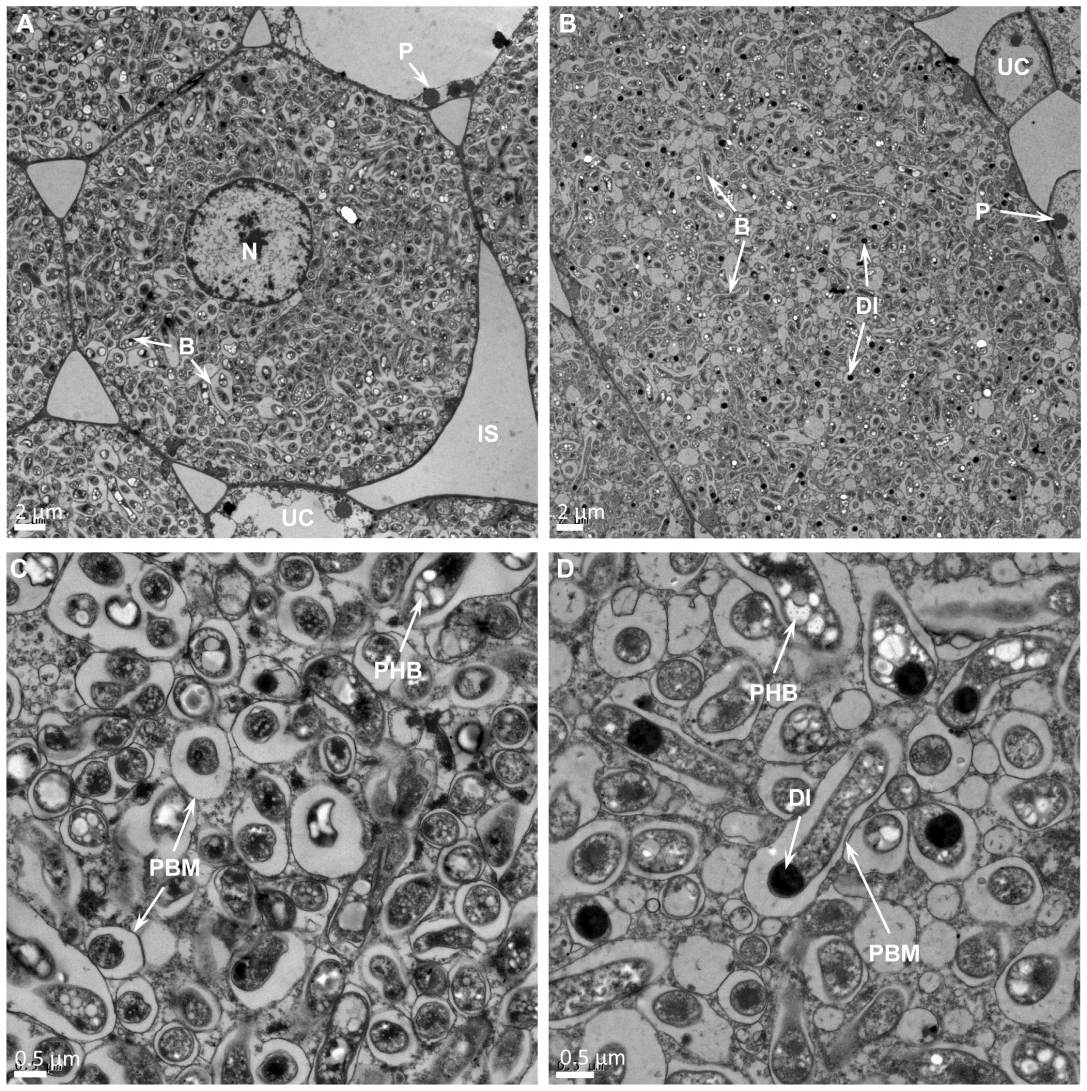
Transmission electron micrographs of *Glycine soja* nodules. Thin sections of 25 DPI nodules were examined by transmission electron microscopy. **(A, B)** In both *Bradyrhizobium diazoefficiens* USDA110 **(A)** and *Bradyrhizobium* sp. strain LVM105 **(B)** elicited nodules, the central infected cells are completely occupied by rhizobia and contain a large number of bacteroids. **(C, D)** Higher magnification view of *B. diazoefficiens* USDA110 **(C)** and *Bradyrhizobium* sp. strain LVM105 **(D)** infected cells reveals numerous symbiosomes that are dilated and contain one or two bacteroids. Note that most bacteroids in LVM105–induced nodules **(D)** accumulate spherical dark inclusions. B, bacteroid; N, nucleus; P, peroxisome; IS, intercellular space; UC, uninfected cell; DI, dark inclusion; PHB, polyhydroxybutyrate; PBM, peribacteroid membrane.

**Figure 4:**
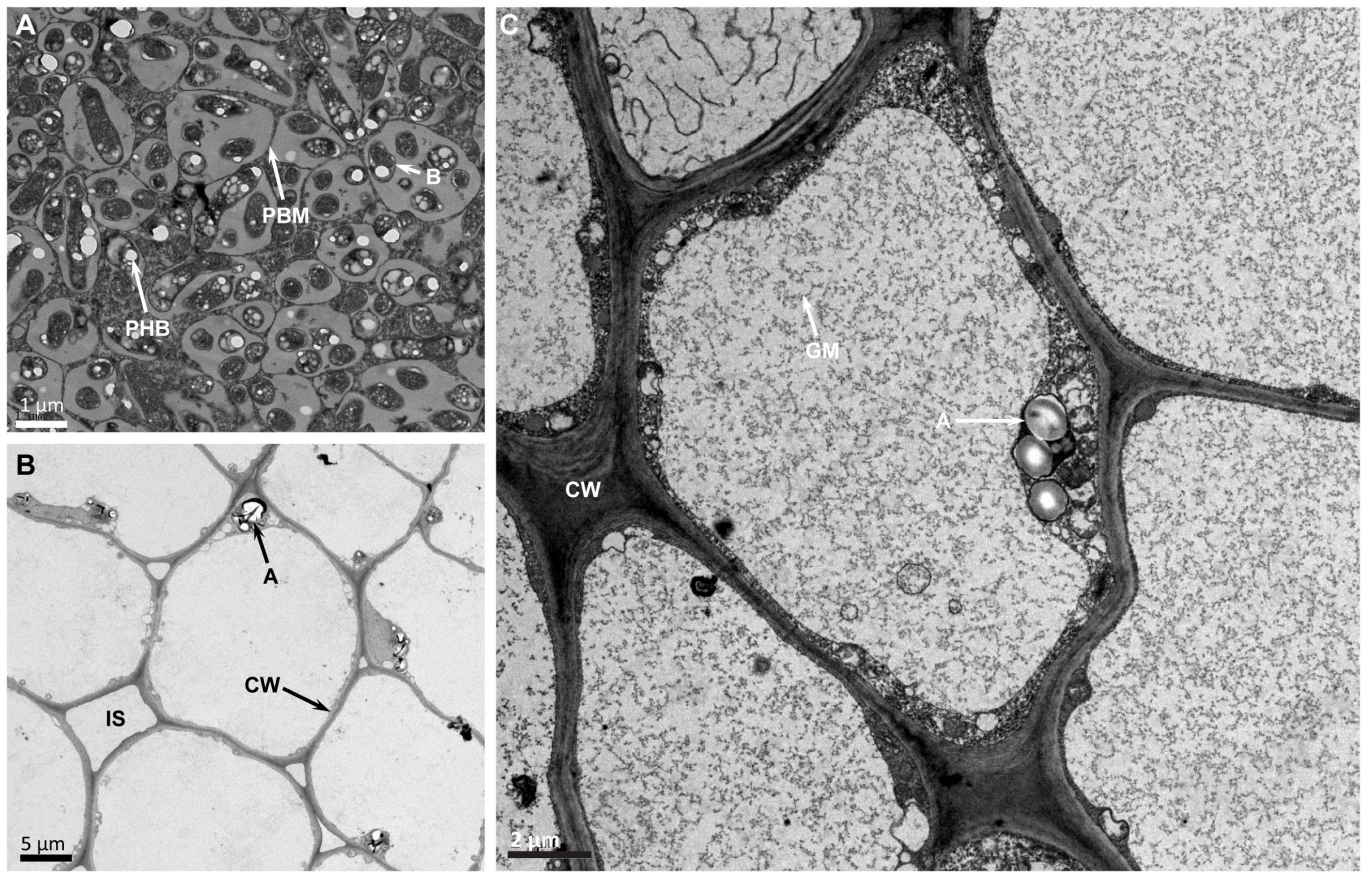
Transmission electron micrographs of *Glycine max* cv. Williams 82 nodules. Thin sections of 25 DPI nodules were examined by transmission electron microscopy. **(A)** Williams 82 nodules elicited by USDA110 reveal cells that are completely occupied by rhizobia and the symbiosomes are enlarged and often contain one or more bacteroids inside them. **(B)** LVM105-elicited “popcorn” nodules contain empty parenchymatous cells that are devoid of any rhizobia. **(C)** Higher magnification view of **(B)** reveals the empty parenchymatous cells contain large central vacuoles, numerous starch grains that can be seen close to the cell walls, and the secondary thickening of the cell walls. B, bacteroid; A, amyloplast; CW, cell wall; GM, granular material; PHB, polyhydroxybutyrate; PBM, peribacteroid membrane.

Transmission electron microscopy observation of thin section of USDA110 formed nodules in *G. soja* clearly demonstrated the presence of cells that were occupied by bradyrhizobia (**Figure 3**). Some of these cells contained a prominent central nucleus (**Figure 3A**). Dark staining spherical structures close to the cell walls were seen only in uninfected cells (**Figure 3A**). The bacteria, which have differentiated into nitrogen-fixing bacteroids were enclosed within plant derived structures called symbiosomes (**Figures 3A, B**). Some symbiosomes enclosed either individual or multiple bacteroids. Some of the bacteroids also contained prominent polyhydroxybutrate crystals (**Figure 3B**). Similar ultrastructural features were also observed in LVM105 formed *G. soja* nodules (**Figures 3C, D**). Interestingly, some of the bacteroids enclosed within the symbiosomes of LVM105 formed nodules contained prominent dark-staining spherical inclusions (**Figure 3D**). The ultrastructure of *G. max* cv Williams 82 nodules induced by USDA110 and LVM105 were also investigated by electron microscopy (**Figures 4A–C**). The anatomy of Williams 82 nodules induced by USDA110 was similar to that observed in the *G. soja* nodules. In the case of Williams 82 nodules, the symbiosomes were dilated and often contained more than one bacteroids (**Figure 4A**). These bacteroids also contained prominent polyhydroxybutrate crystals (**Figure 4**). In contrast, electron microscopy of “popcorn nodules” initiated by LVM105 on Williams 82 revealed the presence of cells that contained prominent central vacuoles (**Figures 4B, C**). Starch grains were located in these cells. Strikingly, these cells contained no bacteria within them and seem to be filled with either granular or fibrillar materials. Additionally, these cells exhibited thickened walls presumably due to secondary wall thickening (**Figure 4C**).

### RNA sequencing-based transcriptome analysis of mature nodules elicited by USDA110 and LVM105 in cultivated and wild soybeans

**Figure 5:**
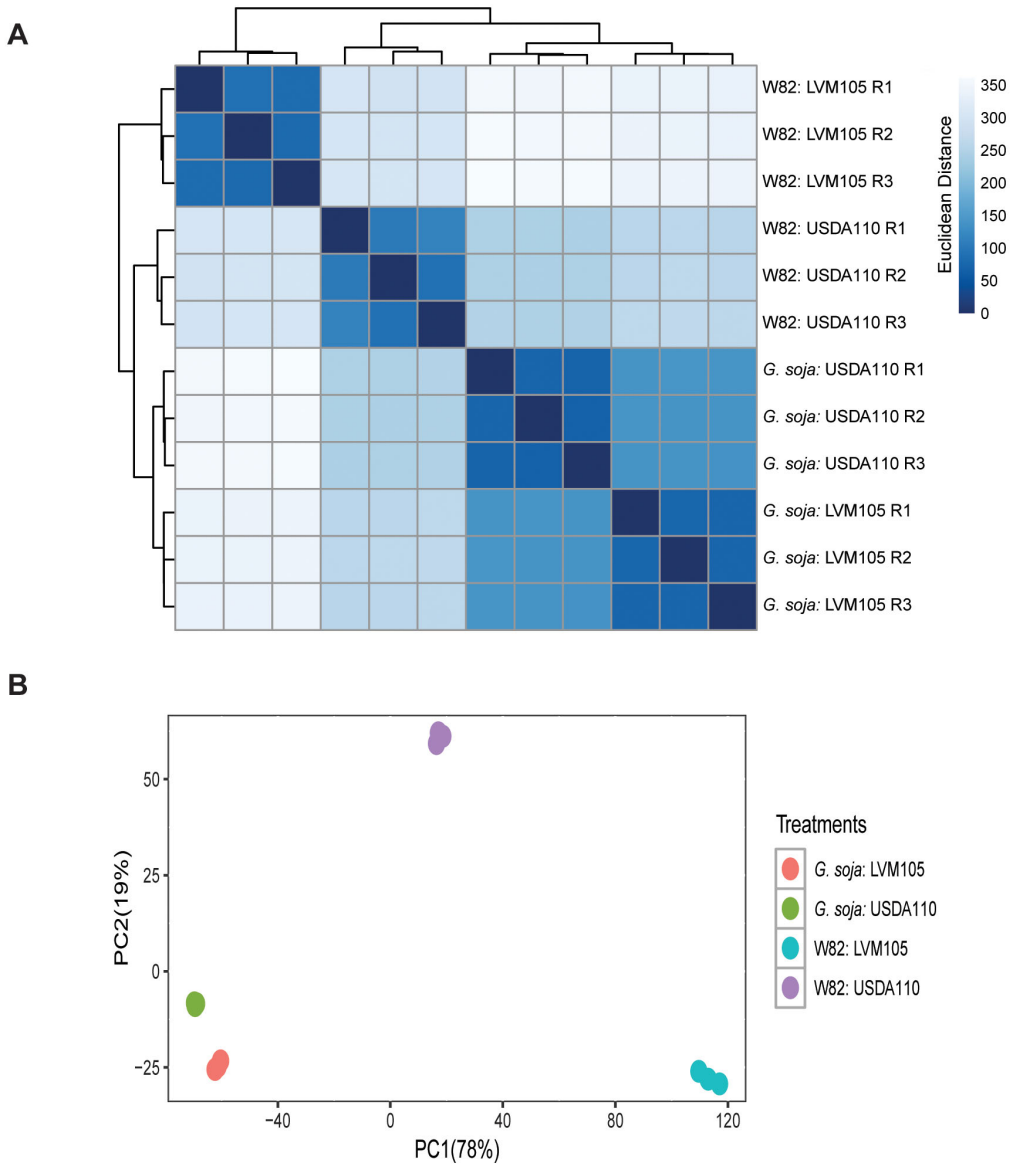
Sample-to-sample distance plot of RNA-seq samples. **(A)** Sample-to-sample distance heatmap showing the Euclidean distances between RNA-seq samples calculated from normalized gene expression count data. Clustering the 12 RNA-seq samples represents the relationships between the four treatments reflected by the intensity of blue color with darker colors indicating closer relationships. **(B)** Principal component analysis (PCA) showing high similarities between the biological replicates of each treatment. The highest degree of variability between non-functional nodules formed on Williams 82 by LVM105 and the functional nodules is demonstrated by the distant clustering on the PC1 axis (78% variance).

To understand the molecular mechanisms controlling soybean response to nodulation by USDA110 and LVM105, we investigated transcriptome reprogramming in 25-day-old nodules induced by USDA110 and LVM105 in Williams 82 and *G. soja* using RNA-seq. Raw sequencing reads from three biological samples for each treatment were mapped to soybean reference genome (Williams 82 version 4) and showed high mapping efficiency ranging from 91.6 to 96.8%. Using normalized read counts we constructed a sample-sample distance heatmap showing the clustering of gene expression profiles of the four treatments (**Figure 5A**). This clustering illustrated the similarity in transcriptome profiles of the functional nodules induced by USDA110 on Williams 82 and *G. soja* and that induced by LVM105 on *G. soja*. Principal component analysis (PCA) of RNA-seq data showed a high similarity between the three biological replicates of each treatment, and similarly separated the popcorn nodules formed on Williams 82 by LVM105 form the functional nodules (**Figure 5B**).

To determine strain and genotype effects, we identified differentially expressed genes (DEGs) in three comparisons. More specifically, we determined DEGs between USDA110– and LVM105–induced nodules in *G. soja* and Williams 82 (*G. soja*: USDA110 vs LVM105 and W82: USDA110 vs LVM105) to determine strain effects. Similarly, we determined DEGs between nodules formed on Williams 82 and *G. soja* and induced by USDA110 (W82/USDA110 vs *G. soja*/USDA110) to determine genotype effects. The USDA110 vs LVM105 comparison in *G. soja* resulted in the identification of 3173 DEGs (1442 upregulated and 1731 downregulated), whereas in Williams 82 this comparison resulted in the identification of 14007 DEGs (6083 upregulated and 7924 downregulated) (**Supplementary Tables 1**, **2**). These data indicate that both strains induce varied gene expression programs during the formation of functional nodules in *G. soja.* The W82/USDA110 vs *G. soja*/USDA110 comparison led to the identification of 9755 DEGs, including 4875 upregulated and 4880 downregulated (**Supplementary Table 3**), reflecting the substantial difference between nodule-associated transcriptomes of cultivated and wild soybeans.

### Impacts of USDA110 and LVM105 on nodule transcriptome in wild soybean

**Figure 6:**
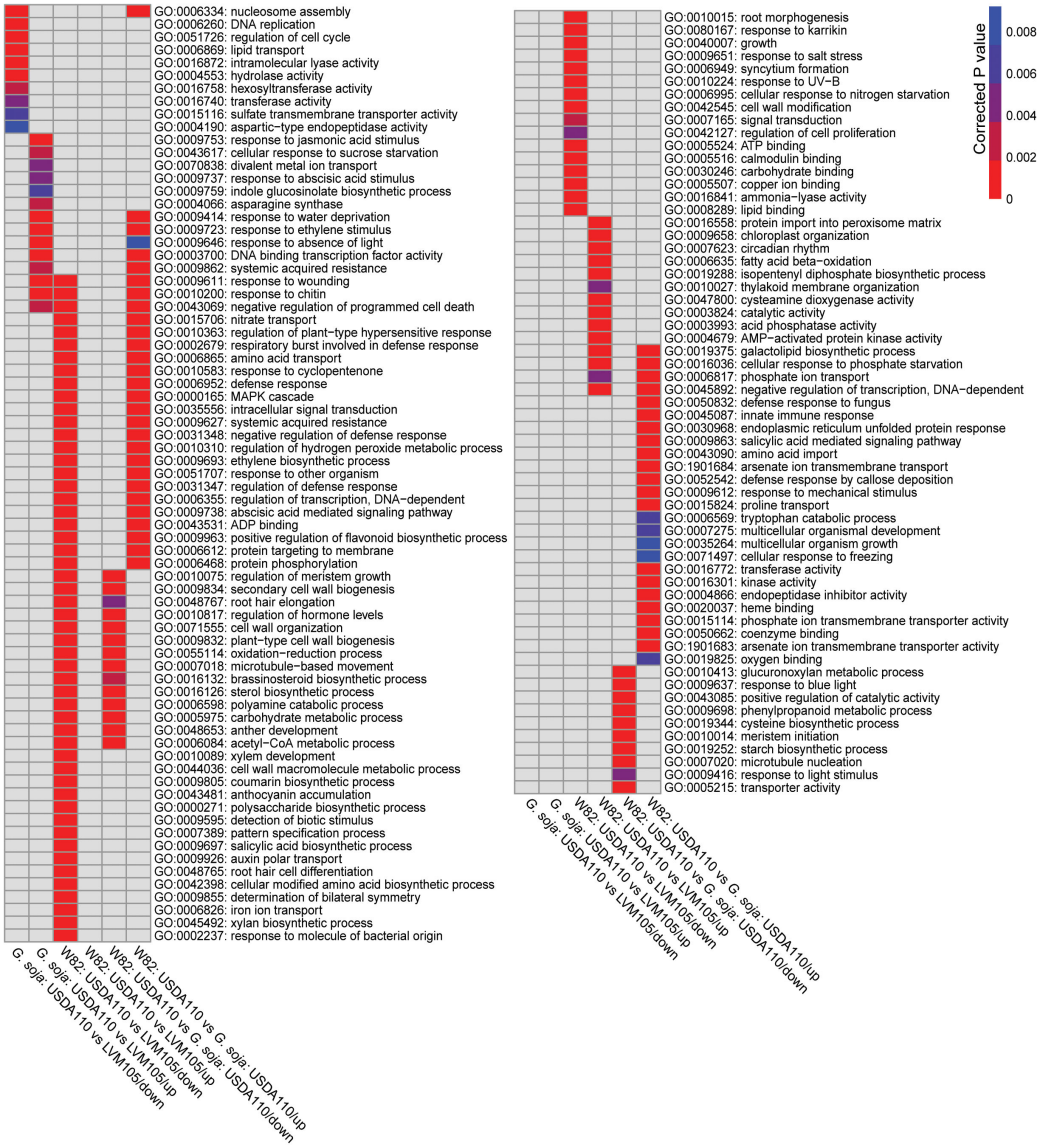
Heatmap representation of significantly enriched gene ontology (GO) terms among the significantly upregulated and downregulated genes in various comparisons. Significantly enriched GO terms among up- and downregulated genes of the indicated comparisons were determined using the GO Term Enrichment Tool at SoyBase (https://soybase.org) with an adjusted P-value less than 0.01 for significance.

**Figure 7:**
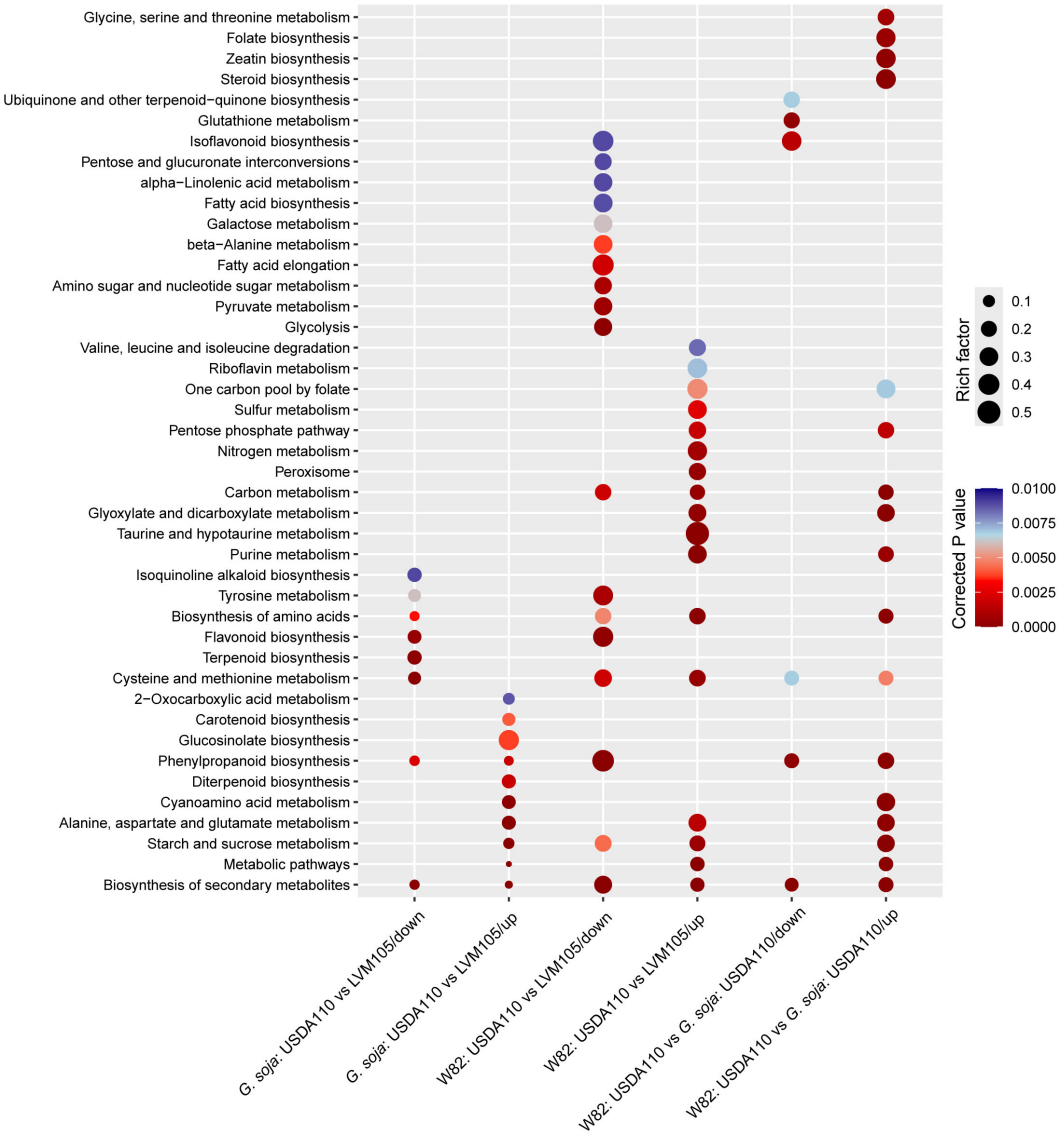
Bubble plot representation of significantly enriched KEGG pathways among the significantly upregulated and downregulated genes in various comparisons. KEGG (Kyoto Encyclopedia of Genes and Genomes) pathway enrichment analysis was performed using KOBAS web server (http://bioinfo.org/kobas) with a corrected P value cut off of 0.01 for significance. Color bar on the right represents the corrected P values. Bubble size represents the rich factor, which is the ratio of the number of significantly upregulated or downregulated genes in a given pathway to the number of background genes annotated in this pathway.

We performed gene ontology (GO) terms enrichment analysis on the DEGs determined in the 3 comparisons mentioned above to associate potential biological processes with the observed nodule phenotypes. The upregulated genes of the USDA110 vs LVM105 comparison in *G. soja* were enriched in genes related to plant response to wounding, response to jasmonic acid and ethylene stimuli, negative regulation of programmed cell death, systemic acquired resistance, indole glucosinolate biosynthetic process, iron ion transport, and transcriptional activity, for example (**Figure 6**). The downregulated genes were enriched in terms related to nucleosome assembly, DNA replication, regulation of cell cycle and lipid transport (**Figure 6**). KEGG pathway analysis revealed enrichment of various pathways among the upregulated genes, including diterpenoid biosynthesis, glucosinolate biosynthesis, and carotenoid biosynthesis (**Figure 7**). The downregulated genes were enriched in pathway related to terpenoid biosynthesis, flavonoid biosynthesis, and biosynthesis of amino acids (**Figure 7**). Notably, the biosynthesis of secondary metabolites and phenylpropanoid biosynthesis pathways were significantly enriched among both upregulated and downregulated genes, reflecting the complex regulation of these pathways in fully-developed nodules. Despite both LVM105 and USDA110 are able to induce the formation of functional nodules in *G. soja*, these biological processes and pathways may contribute to various aspects of symbiotic interactions as well as nodule differentiation, development, and senescence.

### Impacts of USDA110 and LVM105 on nodule transcriptome in cultivated soybean

The upregulated genes of the USDA110 vs LVM105 comparison in Williams 82 were enriched in terms related to galactolipid biosynthetic process, protein import into peroxisome matrix, circadian rhythm, and cysteamine dioxygenase activity, for example (**Figure 6**). Unlike the upregulated genes, a large number of significantly overrepresented GO terms were identified among the downregulated genes. This included, for instance, terms related to protein phosphorylation, regulation of meristem growth, protein targeting to membrane, xylem development, root hair elongation, amino acid transport, secondary cell wall biogenesis, and regulation of hormone levels. Interestingly, among the downregulated genes, we also found several related to defense responses, including regulation of plant-type hypersensitive response, systemic acquired resistance, response to chitin, respiratory burst involved in defense response, MAPK cascade, and detection of biotic stimulus. Thus, upregulation of defense and immunity related genes in the LVM105-indcued non-functional nodules on Williams 82 roots may be contributed to the incompatibility of LVM105–Williams 82 interactions. KEGG pathway analysis indicated that purine metabolism, biosynthesis of amino acids, biosynthesis of secondary metabolites, taurine and hypotaurine metabolism, glyoxylate and dicarboxylate metabolism, and metabolism of nitrogen were the most significantly enriched pathways among the upregulated genes (**Figure 7**). Biosynthesis of secondary metabolites, phenylpropanoid biosynthesis, glycolysis, flavonoid biosynthesis and pyruvate metabolism pathways were the most significantly enriched among the downregulated genes (**Figure 7**).

### Differential nodule transcriptome between cultivated and wild soybean

The differentially upregulated genes between the functional nodules formed on Williams 82 and *G. soja* after inoculation with USDA110 were enriched in genes involved in various aspects of defense responses, including response to chitin, respiratory burst involved in defense response, oxidation-reduction process, regulation of plant-type hypersensitive response, MAPK cascade, and systemic acquired resistance (**Figure 6**). Terms related to negative regulation of defense response and negative regulation of programmed cell death were also found among the upregulated genes (**Figure 6**), indicative of various regulatory signals controlling defense responses during nodulation.

The upregulated gene list was also significantly enriched in genes related to protein phosphorylation, protein targeting to membrane, regulation of flavonoid biosynthetic process, galactolipid biosynthetic process, and signaling pathways mediated by abscisic acid, ethylene, and salicylic acid (**Figure 6**). In addition, various cellular transport processes, including nitrate, amino acid, and phosphate transport were significantly overrepresented among the upregulated genes (**Figure 6**). The most significantly enriched terms among the downregulated genes were those related to regulation of meristem growth, cell wall biogenesis, secondary cell wall biogenesis, oxidation-reduction process, and microtubule-based movement (**Figure 6**). The activity of these biological processes may determine nitrogen fixation capacity in *G. max* and *G. soja*.

KEGG pathway analysis indicated that carbon metabolism, starch and sucrose metabolism, biosynthesis of amino acids, and glyoxylate and dicarboxylate metabolism were significantly enriched among the upregulated genes (**Figure 7**). Glutathione metabolism, isoflavonoid biosynthesis, and biosynthesis of terpenoid-quinones were significantly enriched pathways among the downregulated genes (**Figure 7**). Importantly, biosynthesis of secondary metabolites, cysteine and methionine metabolism, and phenylpropanoid biosynthesis pathways were similarly enriched among the up-and downregulated genes (**Figure 7**), suggesting tight regulation of these pathways during nodulation.

### Differential expression patterns of symbiotic genes in mature nodules elicited by USDA110 and LVM105 in cultivated and wild soybeans

To further understand the molecular mechanisms regulating the symbiotic interactions between soybean and USDA110 and LVM105, we examined the expression patterns of 241 soybean genes known to be required for symbiotic nitrogen fixation (Roy et al., 2020). The expression patterns of these genes in functional nodules induced by USDA110 and LVM105 in *G. soja* were very similar with only 4 genes showing differential expression, suggesting that nodulation signaling pathways induced by USDA110 and LVM105 in mature functional nodules in *G. soja* are quite similar. These genes are involved in rhizobial infection (*Glyma.18G191800* and *Glyma.10G198700*), nodule organogenesis (*Glyma.09G063900*), and symbiosome formation (*Glyma.05G172600*0) (**Supplementary Figure 2A**). However, when the expression patterns of the 241 symbiotic genes were examined in the functional and non-functional nodules elicited, respectively, by USDA110 and LVM105 in Williams 82, a set of 24 genes exhibited differential expression (**Supplementary Figure 2**). Interestingly, the expression of these 24 genes in the functional nodules induced by USDA110 in Williams 82 showed the same patterns in the functional nodules induced by USDA110 and LVM105 in *G. soja* (**Supplementary Figure 3**), suggesting that the activity of these genes is linked to nodule functionality at mature stage. This gene set included several transcription factors, transporters, membrane proteins, oxidoreductases, and kinases, which are involved in various aspects of symbiosis (**Supplementary Figure 3**, **Supplementary Table 4**).

## Discussion

Wild soybean species tends to exhibit greater genetic diversity compared to cultivated soybeans and possess adaptive traits that allow them to grow in nitrogen-poor soils and under other environmental stress conditions. These adaptive traits may enable wild soybeans to associate with a broader range of rhizobia strains compared to cultivated soybeans (Ma et al., 2019). Our data support this view as both USDA110 and LVM105 strains can effectively nodulate *G. soja* but only USDA110 can form effective symbiotic relationships with the cultivated soybean Williams 82. Anatomical comparison between the nodules induced by USDA110 and LVM105 in *G. soja* revealed strikingly similar structural features. However, one key difference lies in the appearance of bacteroids within these nodules. Specifically, LVM105 bacteroids contained a spherical, dark-staining inclusion that was absent in USDA110 bacteroids. Further studies are needed to identify the origins and functions of these spherical inclusions. In contrast to *G. soja*, the nodules formed by LVM105 and USDA110 on Williams 82 exhibited significant structural disparities. While USDA110 induced functional nodules on Williams 82, LVM105 led to the formation of pseudonodules devoid of rhizobia. Notably, in model legumes, cytokinin, a plant hormone, has been shown to be sufficient for pseudonodule induction (Gauthier-Coles et al., 2019). It is plausible that LVM105 inoculation triggers hormonal changes, resulting in specific root cortical proliferation in Williams 82. This possibility is supported with our RNA-seq data showing significant enrichment of various GO terms associated with hormone biosynthetic and signaling pathways among the upregulated genes identified in the pseudonodules as compared with the USDA110-formed nodules in Williams 82 (**Figure 6**). These terms included regulation of hormone levels, auxin polar transport, ethylene biosynthetic process, brassinosteroid biosynthetic process, salicylic acid biosynthetic process, and abscisic acid mediated signaling pathway (**Figure 6**). Furthermore, LVM105-formed nodules on Williams 82 consisted of actively dividing parenchymatous cells containing large central vacuoles. Our ultrastructural analysis revealed prominent secondary cell wall thickening in these cells, which may indicate defense responses. Transcriptome data further support this possibility, showing significant overrepresentation of GO terms related to secondary cell wall biogenesis, cell wall organization and biogenesis, and xylan biosynthetic process among the upregulated genes in LVM105-formed pseudonodules when compared with the functional USDA110-elicited nodules (**Figure 6**).

The broader specificity of wild soybeans to a wide range of rhizobia strains may suggest that *G. soja* undergoes comparable transcriptome reprogramming in response to various rhizobia strains. However, our RNA-seq data revealed that USDA110 and LVM105 induce distinct transcriptome programing in mature nodules formed on *G. soja* roots as exemplified by the identification of 3173 DEGs (**Supplementary Table 1**). While genes involved in nucleosome assembly, DNA replication, regulation of cell cycle, and lipid transport were significantly enriched among the upregulated genes in LVM105*-*induced nodules, genes involved in response to jasmonic acid and ethylene stimuli, negative regulation of programmed cell death, systemic acquired resistance, and indole glucosinolate biosynthetic process were significantly enriched among the upregulated genes in the USDA110*-*induced nodules (**Figure 6**). Nucleosome assembly, DNA replication, and regulation of cell cycle are critical cellular processes for nodule organogenesis and development, which involve complex and coordinated changes in cell division, differentiation, and tissue organization (Foucher and Kondorosi, 2000; Murray, 2016). Cell cycle mechanism therefore appears to play a key role in coordinating these processes to ensure proper development of nodules. The upregulation of defense-related genes as well as jasmonic– and ethylene–responsive genes in the USDA110*-*induced nodules could be a control mechanism to regulate the proliferation and differentiation of cells within the nodules, and hence the balance between accommodating rhizobia and limiting their growth and colonization to support efficient nitrogen fixation. The activation of defense response may also contribute to limiting the proliferation of non-symbiotic bacteria in the rhizosphere without activating full-scale immune responses that could impede symbiotic interactions and nodule functions (Gourion et al., 2015).

The transcriptome comparison between the functional and nonfunctional nodules induced, respectively, by USDA110 and LVM105 in Williams 82 pointed to key biological processes and molecular functions that differentiate between functional and nonfunctional nodules. For example, our data indicate that cell wall biogenesis and organization, xylem development and biosynthetic process, and secondary cell wall biogenesis are activated in non-functional nodules. These processes may simply reflect proliferation and differentiation of cells of the “popcorn nodules” without the formation of infection thread and bacteroids. Alternatively, the activation of genes involved in cell wall biogenesis and organization may constitute part of a defense mechanism that involves strengthening the cell walls to prevent further invasion by non-compatible rhizobium strains and maintain structural integrity. This suggestion is supported by the enrichment of various GO terms associated with defense response among the upregulated genes identified in the LVM105-indcued non-functional nodules in Williams 82.

Our analysis also revealed a downregulation of GO terms related to isopentenyl diphosphate biosynthetic process, AMP-activated protein kinase (AMPK) activity, chloroplast organization, thylakoid membrane organization, and cysteamine dioxygenase activity in non-functional nodules. Plants use the isopentenyl diphosphate biosynthetic process to create isopentenyl diphosphate (IPP) and dimethylallyl diphosphate (DMAPP), which are essential for the biosynthesis of isoprenoids. Isoprenoids are precursors for phytohormones such as gibberellic acids, cytokinins, and abscisic acid, which have been shown to play key roles in root nodulation and nodule organogenesis (Ryu et al., 2012; Breakspear et al., 2014; van Zeijl et al., 2015; Ali et al., 2021; Kildegaard et al., 2021). Besides hormone biosynthesis, isoprenoids also contribute to different aspects of cellular energy production (Giraud and Fleischman, 2004). Therefore, the downregulation of genes involved in the biosynthesis of isoprenoids can have a significant impact on nodulation by limiting cellular energy production and hormone synthesis. Limiting cellular energy in non-functional nodules is not only reflected by downregulation of the biosynthesis isoprenoids but also by the downregulation of AMPKs, which act as the primary sensors of cellular energy status (Hardie, 2011; Garcia and Shaw, 2017). The downregulation of GO terms related to chloroplast organization and thylakoid membrane organization is intriguing despite the fact that nodules typically lack chloroplasts and active photosynthesis. Further studies are needed to elucidate the specific functions of these genes and their contributions to nodule development and function. Considering that protecting cells from oxidative damage and scavenging reactive oxygen species (ROS) are critical for nodule development and function (Becana et al., 2000; Cao et al., 2017; Roy et al., 2020), downregulation of genes involved in cysteamine dioxygenase activity might also contribute to the formation of non-functional nodules. Together, our analysis indicates that activation of genes associated with cell wall biogenesis and organization and various aspects of defense response and immunity together with downregulation of genes involved in the biosynthesis of isoprenoids and antioxidant stress are associated with the formation of non-functional nodules on Williams 82 roots.

Our comparative transcriptome analysis of the functional nodules induced by USDA110 in *G. soja* and Williams 82 pointed to important characteristics of fully-developed nodules in wild and cultivated soybeans. The analysis revealed that genes involved in oxygen binding, amino acid transport, and nitrate transport were significantly enriched and highly expressed in the USDA110-indcued nodules in Williams 82 relative to that formed on *G. soja.* This is consistent with the general trend that nitrogen fixation capacity in various cultivated soybean varieties exceed wild soybeans regardless of the rhizobial strains used (Muñoz et al., 2016). In this context, increased activity of oxygen-binding proteins such as leghemoglobin in the nodules is expected to sustain the symbiotic relationships by creating a microaerobic environment to protect the activity of nitrogenase enzymes from oxygen inhibition, thereby facilitating effective nitrogen fixation capability (Kuzma et al., 1993; Ott et al., 2005; Larrainzar et al., 2020). This anticipated effective nitrogen fixation capability appeared to be associated with increased kinase activity, signal transduction pathways mediated by abscisic acid, ethylene, and salicylic acid, consistent with the demonstrated roles of these signaling pathways in nodule formation and development (Lin et al., 2020; Roy et al., 2020; Luo et al., 2023). The activation of defense and immunity genes in fully-developed and functional nodules may seem counterintuitive considering that nodules are sites of symbiotic interactions. However, the establishment and maintenance of symbiotic interactions may require active regulation of plant defense responses in order to monitor and control the microbial populations within the nodules and prevent secondary infection, thereby ensuring the integrity and functionality of nodules for nitrogen fixation (Gourion et al., 2015). Additionally, there is growing evidence of cross-talk between nodulation signaling pathways and defense signaling pathways mediated by ethylene and salicylic acid (Lopez-Gomez et al., 2012; Zipfel and Oldroyd, 2017; Yu et al., 2019; Roy et al., 2020), and this cross-talk may fine-tune the symbiotic interactions and optimize nodule development and nitrogen fixation activity. KEGG pathway analysis also revealed that differential expression of highly enriched genes related to the biosynthesis and metabolism of various amino acids, zeatin biosynthesis, isoflavonoid biosynthesis, glyoxylate and dicarboxylate metabolism, folate biosynthesis, and starch and sucrose metabolism may also contribute to differences in symbiotic nitrogen fixation between wild and cultivated soybeans given the reported functions of these pathway in establishing the symbiotic interactions in various leguminous plants (Prell and Poole, 2006; Subramanian et al., 2007; Gamas et al., 2017; Roy et al., 2020; Lyu et al., 2022; Piya et al., 2023).

Analyzing the expression patterns of 241 soybean genes known to be essential for symbiosis (Roy et al., 2020) revealed that nodulation signaling pathways induced by USDA110 and LVM105 in mature functional nodules in *G. soja* are comparable. Only 4 genes encoding CYTOKININ OXIDASE (Glyma.09G063900), RHIZOBIUM-DIRECTED POLAR GROWTH (RPG, Glyma.10G198700), ACTIN RELATED PROTEIN (Glyma.05G172600), and NODULIN 5 (Glyma.18G191800) showed distinct expression in the functional nodules induced by USDA110 and LVM105 in *G. soja*. Despite the limited number, the reported functions of these genes in various aspects of symbiosome and nodule development (Arrighi et al., 2008; Pii et al., 2012; Gavrin et al., 2015; Reid et al., 2016) suggest that genetic differences in Rhizobium strains can influence the expression of symbiotic genes with functions related to nodule development and nitrogen fixation.

Our analysis also resulted in the identification of 24 symbiotic genes that can differentiate between functional and nonfunctional nodules. Among these genes we found three KNOX homeodomain transcription factors (*Glyma.06G065200*, *Glyma.04G064100*, and *Glyma.14G112400*), and the CYTOKININ OXIDASE 3 (*Glyma.15G140000*), which function in nodule development (Di Giacomo et al., 2017; Reid et al., 2016). The gene list also included *SYNAPTOGAMIN* (*SYT1/2/3*, *Glyma.10G210000*), *SYMBIOTIC REMORIN1* (*SymREM*, *Glyma.08G012800*), *SYNTAXIN OF PLANTS 132* (*SYP132*, *Glyma.13G307600*), and the *REGULATOR OF SYMBIOSOME DIFFERENTIATION (RSD*, *Glyma.07G135800*), which regulate symbiosome differentiation (Lefebvre et al., 2010; Ivanov et al., 2012; Sinharoy et al., 2013; Gavrin et al., 2017). Homologs of genes required for bacterial maturation (*Glyma.08G076300*, SEN1), and nodule transport and metabolism (*Glyma.07G088200*, SST1) were also identified (Krusell et al., 2005; Hakoyama et al., 2012). Our finding that the expression levels of these transcription regulators were markedly lower in non-functional nodules compared to functional nodules implies that high activity of these genes are required for fully operative nodules. In contrast, the increased expression of genes encoding CHALCONE REDUCTASE (CHR, Glyma.02G307300), ROOT DETERMINED NODULATION 1 (RDN1, Glyma.02G279600), BRASSINOSTEROID INSENSITIVE 1 (BRI1, Glyma.04G218300), NODULE-SPECIFIC PLAT DOMAIN PROTEIN 2 (Glyma.15G008800), FERRITIN 3 (FER3, Glyma.03G050100), and MOLYBDATE TRANSPORTER (MOT1.3, Glyma.14G130000), at the later stage of nodule development may be linked to pseudonodule formation.

In conclusion, our structural and gene expression analyses provided novel insights into the molecular mechanisms controlling nodule functionality and development in cultivated and wild soybeans. The complexity of the underlying mechanisms is reflected by the expression changes of thousands of genes encoding structural proteins, signal transduction proteins, transcriptions factors, and enzymes involved in a wide range of cellular processes, including growth, development, differentiation, metabolism, and defense and stress responses. Our data also indicate that different Rhizobium strains may have evolved to optimize their symbiotic relationship with specific host plants. This evolutionary adaptation can lead to variations in the expression of symbiotic genes to enhance the efficiency of nitrogen fixation and nodule function.

## Data Availability

RNA-seq data described in this study are available at the NCBI database (www.ncbi.nlm.nih.gov/genbank), Gene Expression Omnibus under accession number GSE267634.
