# Supplementary figures and images for: Differential symbiotic compatibilities between rhizobium strains and cultivated and wild soybeans revealed by anatomical and transcriptome analyses
Source: Front Plant Sci. 2024 Sep 3;15:1435632. doi: 10.3389/fpls.2024.1435632 (PMC11405202; doi:10.3389/fpls.2024.1435632)

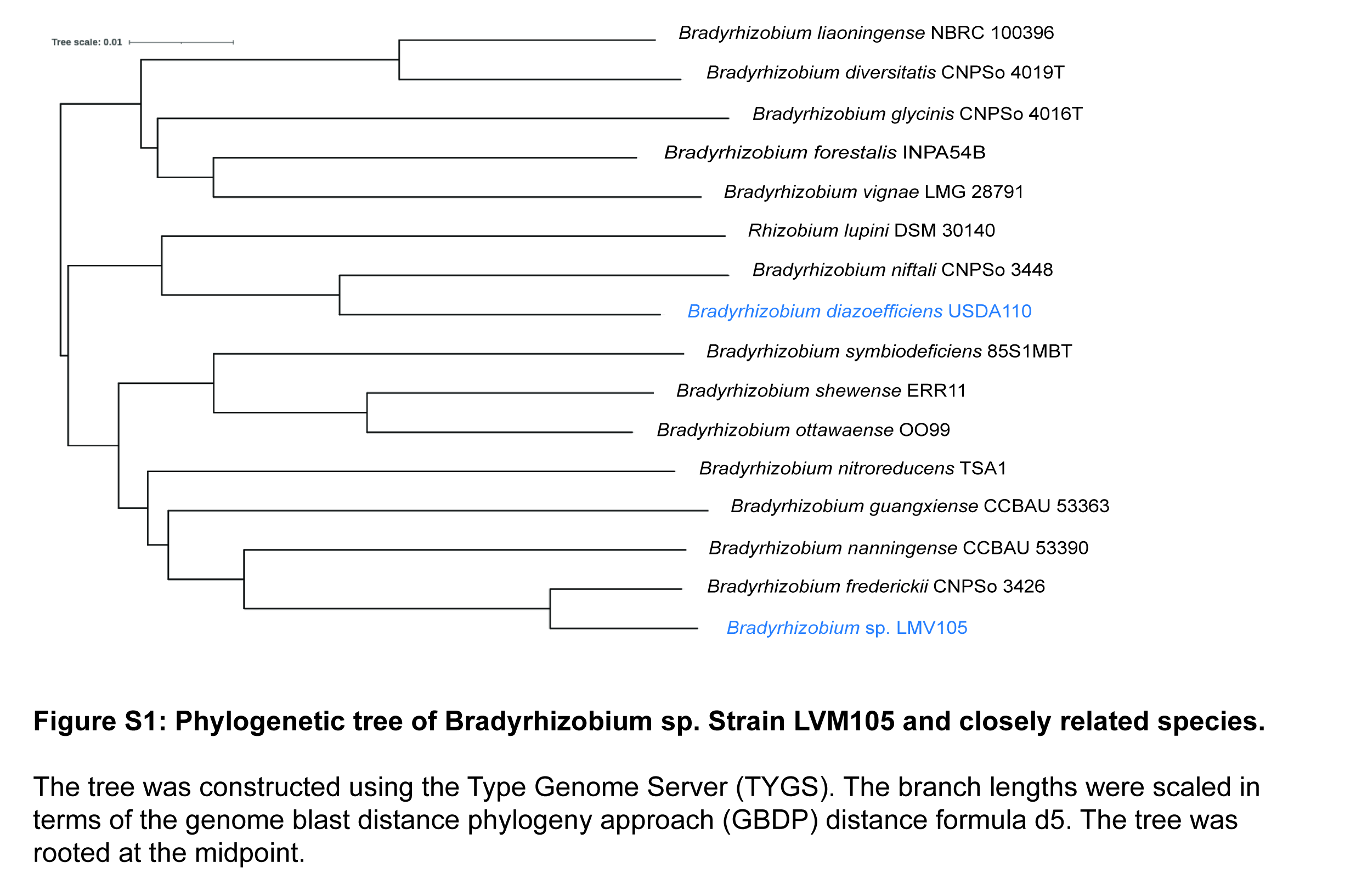

Supplement: Supplementary file 2 [file Image1.tif]

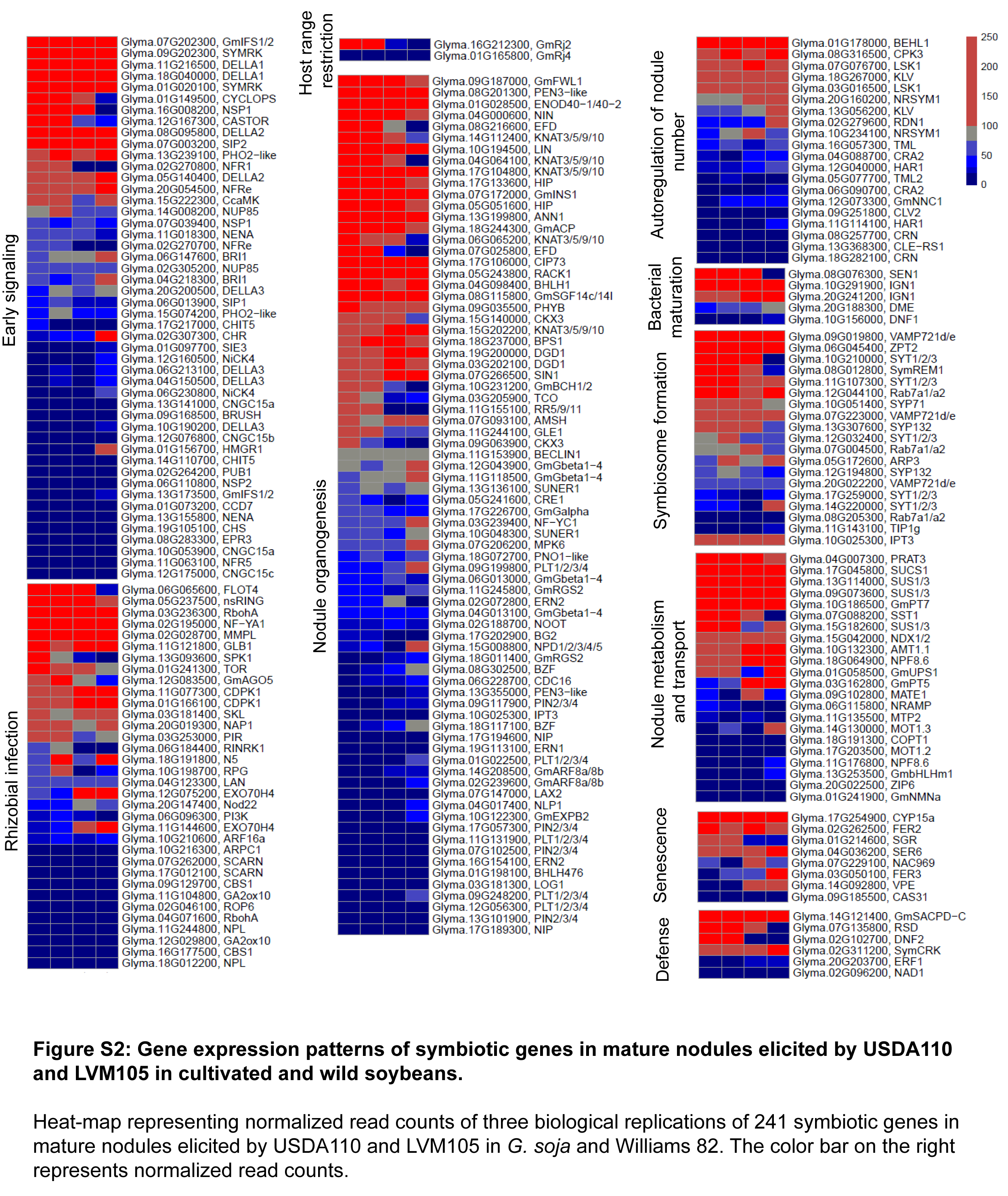

Supplement: Supplementary file 3 [file Image2.tif]

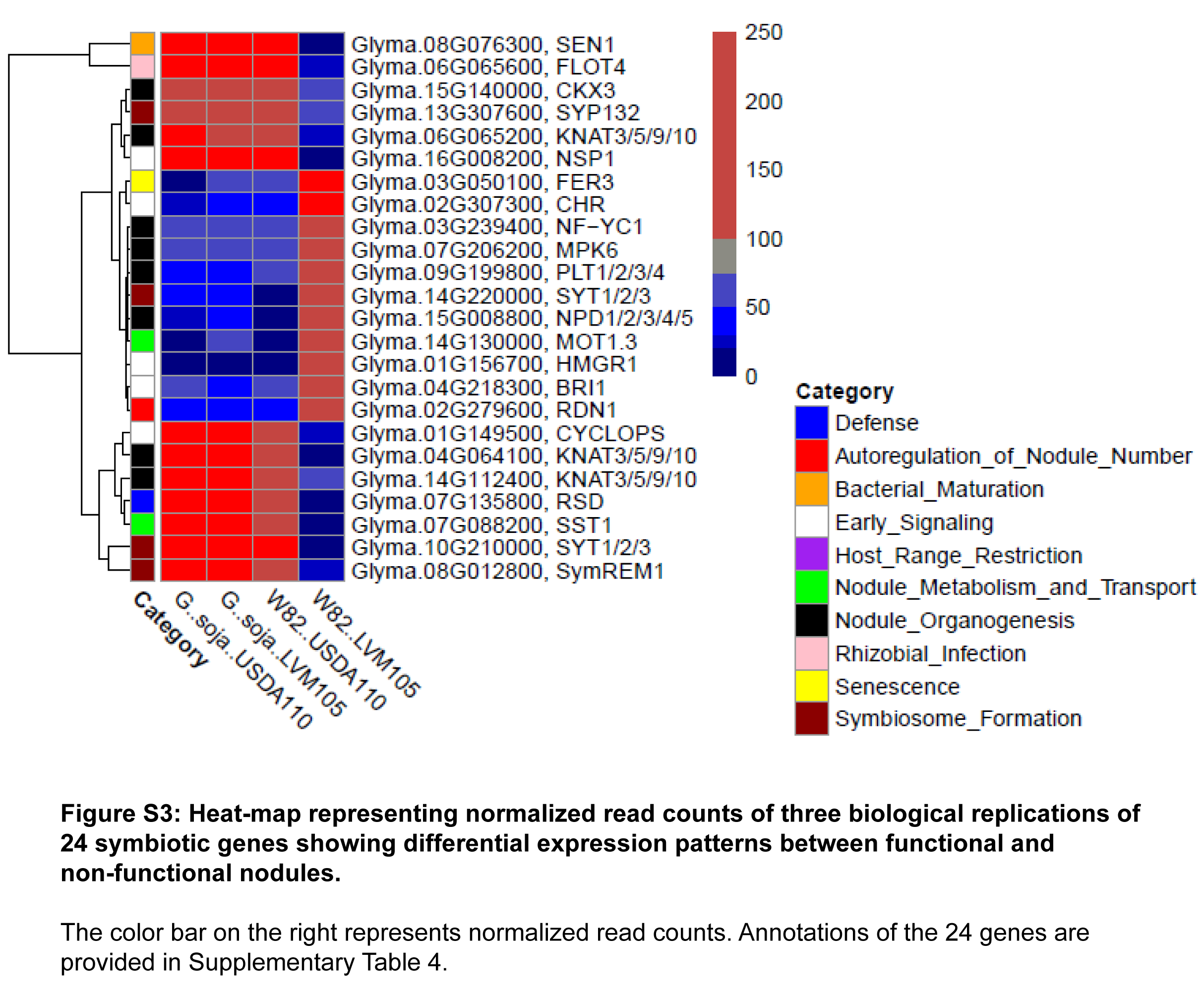

Supplement: Supplementary file 4 [file Image3.tif]
